# Morphological Distinctiveness and Phylogenetic Analysis of *Strobilanthes malvifolia* (Acanthaceae), a New Species From Dry‐Hot Valley in Yunnan, Southwest China

**DOI:** 10.1002/ece3.73093

**Published:** 2026-02-16

**Authors:** Huai‐Yu Chen, Feng Yang, Shao‐Yun Liu, Ting‐Ting Wang, Huan‐Chong Wang

**Affiliations:** ^1^ School of Ecology and Environmental Science Yunnan University Kunming China; ^2^ Herbarium of Yunnan University Kunming Yunnan China; ^3^ State Key Laboratory of Vegetation Structure Function and Construction (VegLab) Kunming Yunnan China

**Keywords:** dry‐hot valley, endemism, phylogeny, plastid genome, *Strobilanthes*

## Abstract

The genus *Strobilanthes* (Acanthaceae) is known for its high morphological diversity and taxonomically challenging. During field investigations in Yunnan, southwest China, we discovered a remarkable new species, *Strobilanthes malvifolia* Huan C. Wang & Huai Yu Chen, which is described and illustrated here. This species exhibits distinct morphological characteristics, including cordate leaves with relatively long petioles (up to 8.7 cm long), sparsely and irregularly rounded serrations along the leaf margin. The complete chloroplast genome of this new species was also sequenced, assembled, and annotated. It is endemic to the Jinsha River Basin in western Yunnan, and was discovered in the dry‐hot valley characterized by extreme aridity and high temperatures. Phylogenetic analyses based on nrITS sequences support its placement as sister to *S. japonica* within the Chamiponella group. Palynological examination reveals spherical, tricolporate pollen with verrucate exine sculpturing arranged in rows, consistent with the pollen type found in Chamiponella group. The species has a very restricted distribution and a small population, with no protection in any nature reserve. According to the IUCN Red List Categories and Criteria, the conservation status of this new species has been preliminarily assessed as Endangered (EN).

## Graphical abstract

This article describes a new species *Strobilanthes malvifolia* (Acanthaceae), endemic to the dry‐hot valleys of the Jinsha River basin in Yunnan, southwest China, highlighting its distinctive cordate leaves with long petioles and irregularly crenate margins. Phylogenetic analyses based on nrITS and plastid trnL‐trnF regions, along with palynological evidence, place it as a sister species to *S. japonica* within the Chamiponella group, and it is proposed to be preliminarily classified as Endangered (EN) following the IUCN Red List criteria due to its extremely restricted distribution and small population size.

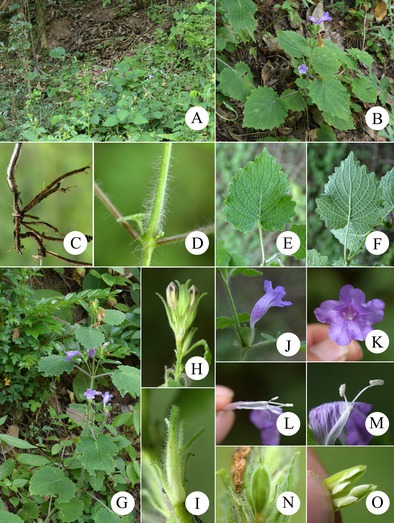

## 1 Introduction

*Strobilanthes* Blume (Ruellieae: Strobilanthinae) is one of the most diverse genera in the family Acanthaceae (Moylan et al. 2004; Tripp et al. 2022). It comprises approximately 450 species, mainly distributed in the tropical and subtropical regions of Asia, with some species extending to the Pacific Islands (Tripp et al. 2013; Deng 2019; Mabberley 2017). *Strobilanthes* is unique in having rugulae and trichomes that retain the style on the inner surface of the posterior corolla tube, and stamens monadelphous (by a sheath) at the base (Hu et al. 2011; Kladwong and Chantaranothai 2024). Some species in the genus are important medicinal plants, such as *S. cusia* (Nees) O. Kuntze, *S. crispa* (L.) Blume, *S. ciliata* Wall. ex Nees (e.g., George et al. 2017; Hu et al. 2011; Mabberley 2017; Ng et al. 2021; Zhu et al. 2022). In addition, some taxa also have significant value in ornamental use, dye production, and as beverages (Kumar et al. 1998; Zhang et al. 2021; Hua et al. 2022; Wood and Adhikari 2014).

Due to the highly variable morphological characteristics and rich species diversity, there has long been significant divergence in the circumscription of the genus since its establishment by Blume in 1826 (McDade et al. 2008). This has led to debate over whether the genus should be adopted with a broad or narrow circumscription. Anderson was the first to use ovule number, an anatomical feature, to separate morphologically similar species of this genus (Anderson 1867). Lindau (1895) applied pollen characteristics to the classification of the Acanthaceae and defined the genus *Strobilanthes* as having the “Rippenpollen” type, characterized by ellipsoidal pollen grains with ridges. Since then, pollen morphology has become an important basis for classification and has played a crucial role in distinguishing genera. Bremekamp (1944) divided *Strobilanthes* sensu Anderson (1867) into 54 smaller genera using combinations of macromorphological, anatomical, and pollen characters. However, many researchers have avoided Bremekamp's classification because the generic boundaries within subtribe Strobilanthinae remained ambiguous (Terao 1983; Wood 1994, 1995, 1998; Wood and Scotland 2003; Wood et al. 2003; Carine and Scotland 2002). Molecular studies (Moylan et al. 2004) have shown that most of these segregate genera in Bremekamp's classification are non‐monophyletic, and a single expanded genus, *Strobilanthes* sensu lato, is proposed at the level of the well‐supported monophyletic subtribe Strobilanthinae. Thereafter, the broad sense *Strobilanthes* has been generally accepted (Deng et al. 2006; Wood and Scotland 2009; Hu et al. 2011; Wood 2014; Deng 2019).

The genus *Strobilanthes* exhibits high diversity in China, which is one of the major distribution areas and centers of diversification for the genus. Hu and Cui (2006) adopted Bremekamp's classification for the systematics and taxonomy of taxa occurring in China, recording 114 species within the subtribe Strobilanthinae, belonging to 23 genera in *Flora Reipublicae Popularis Sinicae*. In the recent treatment of *Flora of China*, Hu et al. (2011) adopted the *Strobilanthes* sensu lato approach and recognized 128 species in China, including 57 endemics. Most of these species are distributed in the southwestern and southeastern China, with Yunnan Province having the highest species richness. As early as 1984, Wu adopted Bremekamp's classification in *The catalog of seed plants in Yunnan Province*, where he recorded 61 species in Yunnan, placed within 12 genera, and proposed new taxonomic combinations for three species (Wu 1984). Hu and Cui (2006) followed this classification framework in *Flora Yunnanica*, recording 72 species within 21 genera of the subtribe Strobilanthinae in Yunnan. In recent years, several new species have been reported (e.g., Chen et al. 2019, 2020; Deng, Wood, and Fu 2010; Deng, Wood, and Li 2010; Thanh et al. 2018; Thomas et al. 2018, 2019, 2020; Wood et al. 2019, 2022), leading to ongoing updates to the species checklist. According to the *Catalogue of Life China 2025 Annual Checklist*, a total of 136 species, 4 varieties, and 1 subspecies of *Strobilanthes* have been recorded from China to date (CBCAS 2025).

During investigations and research on plant diversity in Yunnan, southwestern China, we discovered a plant that differs markedly from known species of *Strobilanthes* in its leaf morphology. Through detailed observations of living individuals in the field, comparative analysis of herbarium specimens, morphological measurements in the laboratory, and thorough review of relevant literature (Wood 1994; Hu and Tsui 2002; Hu and Cui 2006; Karthikeyan et al. 2009; Hu et al. 2011; Adhikari 2018; Wood et al. 2022; Roy et al. 2023; Kladwong and Chantaranothai 2024), we have preliminarily determined that this plant represents a previously undescribed new species. In this study, we provide a comprehensive morphological description of the new species and discuss its systematic position within the genus based on molecular phylogenetic analyses and palynological evidence. Additionally, we present preliminary data on its geographic distribution, habitat preferences, and conservation status, offering new insights for future taxonomic and evolutionary studies of the genus.

## 2 Materials and Methods

### 2.1 Morphological Analyses

The morphology of the new species was studied based on observation of living plants and specimens housed at YUKU. Digital images of the genus available at online databases, including the JSTOR Global Plants (http://plants.jstor.org/), the Global Biodiversity Information Facility (GBIF, https://www.gbif.org/), the Indian Virtual Herbarium (https://ivh.bsi.gov.in/), and the Chinese Virtual Herbarium (CVH, https://www.cvh.ac.cn/), as well as collections housed at CDBI, IBSC, KUN, PE and YUKU, were extensively examined and compared with the new species. The herbarium abbreviations follow the Index Herbariorum (Thiers 2025, continuously updated). Pertinent taxonomic literature was extensively consulted (Wood 2014). Field trips were conducted in Dayao County, Yunnan to sample the new species. Images of the plants and flowers of the new species were obtained in the field using a D2X digital camera (Nikon, Tokyo, Japan), and all parts of the flowers were detached and photographed. Specimens with flowers were collected and preserved using both dry and wet methods (Jain and Rao 1977). For the morphological measurements and observations of this new species, the materials used in this study were primarily derived from three independent field gatherings (*S. Y. Liu et al. DY23106*; *S. G. Li et al. DY23333*; *W. M. Zhu et al. 02557*), comprising a total of 11 specimen sheets. The observations covered all morphological characters, including roots, stems, leaves, flowers, fruits, and seeds. The measured traits included plant height, leaf length, leaf width, petiole length, inflorescence length, pedicel length, bract length and width, bracteole length, calyx length, corolla length, and fruit length. All morphological observations and measurements were conducted using a stereomicroscope (Olympus SZX2, Tokyo, Japan), with a ruler (precision: 1 mm) or a metric vernier caliper (precision: 0.02 mm).

### 2.2 Palynological Studies

Mature pollen grains of the new species were sampled from the type materials and attached directly to carbon adhesive tape. They were coated with gold–palladium using a BAL‐TEC SCD 005 cool sputter coater (BAL‐TEC AG, Liechtenstein) at Yunnan University, Kunming, China. Observations were conducted using a Nova NanoSEM450 scanning electron microscope (SEM) (FEI Co., USA) at 10 kV at a magnification of 3500×. Descriptive terminology for the pollen grains follows Punt et al. (2007). Pollen dimensions, including polar axis (P) and equatorial axis (E), were measured using the updated software tool MATO (Liu et al. 2023).

### 2.3 Material Sampling, DNA Extraction, Sequencing, nrDNA and Plastome Assembly, and Annotation

Samples of the new species for molecular analysis were collected from Sanchahe Town, Dayao County, Yunnan Province, China. Samples and vouchers were deposited in the herbarium at Yunnan University (YUKU). The genomic DNA was extracted from silica gel‐dried leaves using the modified CTAB method (Doyle and Doyle 1987). The short‐insertion library (350 bp) was constructed and then sequenced to obtain 2 × 150 bp paired‐end data by using the Illumina NovaSeq 6000 platform (Illumina, San Diego, CA, USA) at Novogene (Beijing, China). The 3G of raw data for the new species was filtered through Trimmomatic v. 0.39 (Bolger et al. 2014) to obtain clean data, and then the clean data was quality‐controlled using FastQC v. 0.11.9 (Simon 2010). The nrDNA data and plastid genome were assembled using GetOrganelle v. 1.7.5 (Jin et al. 2020). The plastid genome was annotated using CPGAVAS2 (Shi et al. 2019) and Geseq (Tillich et al. 2017). The plastome maps were drawn using the Organellar Genome DRAW tool (Greiner et al. 2019).

### 2.4 Phylogenetic Analysis

To investigate the phylogenetic position of this species, two DNA sequences, including ITS and *trn*L‐*trn*F, were selected as phylogenetic markers in this study based on Fernandes et al. (2025). The ITS region was extracted from assembled nrDNA, and other ITS sequences are those used by Fernandes et al. (2025) and from NCBI database. There are a total of 74 taxa of *Strobilanthes* to be used, representing most major clades within the genus. In addition, *Ruellia brevifolia* (Pohl) C. Ezcurra, *Hygrophila corymbosa* (Blume) Lindau, and *Sanchezia speciosa* Leonard were selected as outgroups. A total of 70 taxa with 3 outgroups were included in the *trn*L‐*trn*F dataset. The *trn*L‐*trn*F plastid marker was extracted either from the annotated plastid genome or downloaded from GenBank. The voucher specimens and GenBank accession information of the molecular markers used in this study are listed in Table A1.

Due to the limited number of identical species between the two datasets, and most of the identical species are not from the same individual, a combined dataset analysis was not conducted. All sequences were aligned using MAFFT (Katoh and Standley 2013), and the phylogenetic tree was constructed using Phylosuite v.1.2.1 (Zhang et al. 2020; Xiang et al. 2023). Maximum Likelihood (ML) and Bayesian Inference (BI) methods were used to reconstruct phylogenetic trees. The ML analyses were conducted using IQ‐TREE (Nguyen et al. 2015; Minh et al. 2013; Guindon et al. 2010), ML bootstrap values (ML_BS_) were calculated applying 1000 bootstrap replicates with the substitution model chosen according to AIC. BI analysis and posterior probability (BI_PP_) calculation were also conducted in PhyloSuite v.1.2.1 using MrBayes (Ronquist et al. 2012). The best‐fit model for BI analysis was chosen according to BIC using ModelFinder (Kalyaanamoorthy et al. 2017).

## 3 Results

### 3.1 Morphological Characters

*Strobilanthes malvifolia* possesses distinctive leaf morphology within the genus. Its leaves are cordate with a length‐to‐width ratio close to 1:1, opposite and nearly equal in size, and with relatively long petioles, reaching up to 8.7 cm. The leaf margins are sparsely and irregularly crenate (Figure 4K). These characteristics clearly distinguish it from other species within the genus. A comparative analysis was conducted between the new species and the phylogenetically closest taxa ( *S. japonica* and *S. galeopsi*s), as well as the morphologically more similar taxon ( *S. attenuata* ), as shown in Table 1. For a detailed morphological description, see Section 5.3 Taxonomic Treatment.

**TABLE 1:** Morphological comparison among *Strobilanthes malvifolia, S. japonica, S. galeopsis,* and *S. attenuata* .

Characters	Species			
	*S. malvifolia*	*S. japonica*	*S. galeopsis*	*S. attenuata*
Habit	Herbaceous	Herbaceus	Herbaceous	Herbaceous
Stems	Erect, somewhat flexuose, crinite and pilose	Erect, nearly glabrous	Erect fulvo‐tomentellus	Erect, hirsute, setae
Petioles	1–8.7 cm	0.3–1.5 cm	2.5–3 cm	2.5–8.5 cm
Leaves dimension	4–11 × 3.5–10 cm	2–5 × 0.5–2 cm	6–9 × 3–6 cm	4.5–8 × 8–16 cm
Leaf shapes	Cordate	Elliptico‐lanceolata	Ovate	Ovate
Leaf bases	Cordate	Attenuata	Rotundata or brevissime contracta	Cordate
Leaf margins	Irregularly crenate	Entire or slightly repand	Dentato‐crenata	Serrata, serraturis incurvis
Leaf apices	Acuminate	Slightly obtuse and attenuate	Caudato‐acuminata	Caudato‐acuminata
Veins	Crinite, 4–7 pairs	Glabrous, 3–5 parirs	Setulosa, 7–8 pairs	Boary‐pubesceut, 4–8 pairs
Bracteses	Lanceolato, pubescent	Imbricate, spatulate‐obovate, ciliate	Lanceolato‐oblongae, setosae	Lanceolato‐linearis, glanduloso‐hirsutae
Braeteoles	Linear, glandular and pubescent	Linear, obtuse	Linear, calyce paulobreviores	Linear, glanduloso‐hirsutae
Calyces	Linear, glandular and Pubescent, segmenta subzequalia	Linear, scabrid‐puberulent, upper lobe longer	Lanceolato‐linearia, hirsuta and setulosa, segmenta subzequalia	Lineares, hirsutis‐glandulosis, laciniisupera longiore
Corollas	1.7 cm, light purplish‐red, funnelform, short pubescence, glandular‐pilose pubescence	1.5 cm, favescens, parum obliqua, campanulate‐cylindric	2.6 cm, albus to violaceus, extus parce pilosulus	2 cm, purpurea, glabra
Styles	Pilose	Hirtello‐puberus	Pilosula	Pubescent
Filamentums	Pubescent	Sparsely pilosulous	Pilosula	Sparsely pubescent
Seeds	4, obliquely ovoid	4, ovate	4, ovato‐orbieularia	4, linear‐oblong
References	/	Miquel (1865)	Stapf (1894)	Jacq. ex Nees (1847)

### 3.2 Pollen Morphology

**FIGURE 1:**
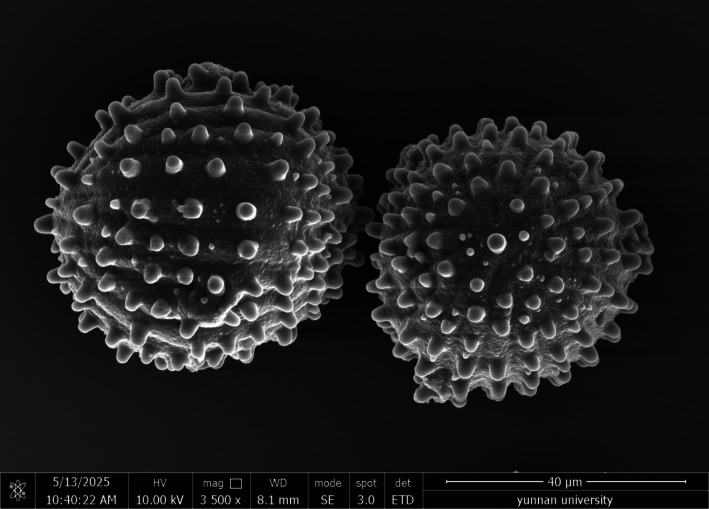
Pollen morphology of *Strobilanthes malvifolia* sp. nov. observed under SEM. The left one shows the equatorial view, and the right one shows the polar view.

The pollen grains of *Strobilanthes malvifolia* are globose in shape (Figure 1), 57.65 (57.1–58.2) × 52.3 (51.2–53.4) μm, P/E = 0.79. They are circular in polar view, with radial symmetry, possessing three colpi alternating with pseudocolpi; the exine surface bears wart‐like verrucae of varying sizes, arranged in distinct or indistinct rows.

### 3.3 Characteristics of the Complete Plastid Genome and nrDNA

**FIGURE 2:**
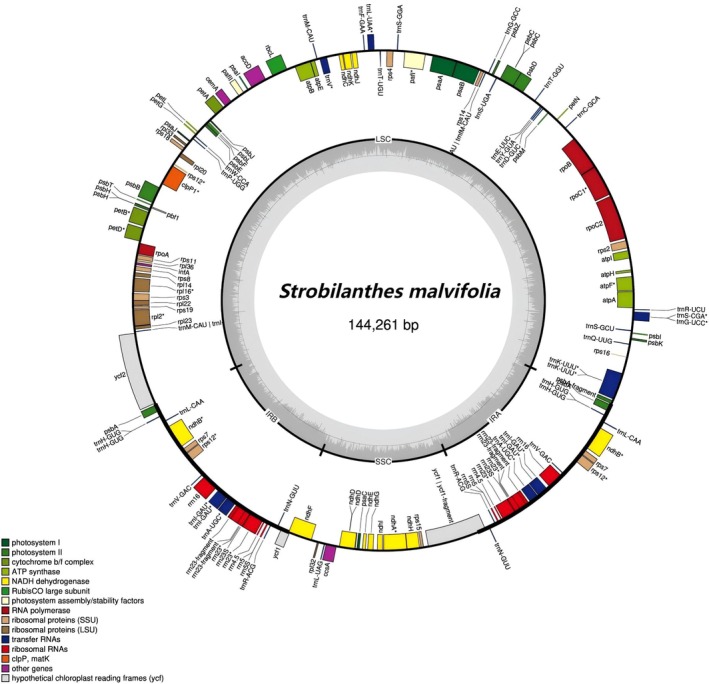
Chloroplast genome of *Strobilanthes malvifolia* sp. nov. Genes on the outside and inside of the circle are transcribed in the clockwise and counterclockwise directions, respectively. The dark and light gray bars in the inner circle denote G + C and A + T contents, respectively. The IRA and IRB (two inverted repeating regions); LSC (large single‐copy region); and SSC (small single‐copy region) are indicated outside of GC content.

The complete plastid genome sequence of *Strobilanthes malvifolia* is 144,261 bp in length. It features a circular and typical quadripartite structure, and contains a large single‐copy region (LSC, 52,574 bp), a small single‐copy region (SSC, 17,738 bp), and two reverse sequence repeat regions (IR, 36,975 bp each) (Figure 2). The percentage of GC in the whole genome is 38.3%, and the corresponding values in LSC, SSC, and IR regions are 34.7%, 30.2%, and 42.9%. We recovered a total of 129 functional genes, including 84 protein‐coding genes, 37 tRNA genes, and 8 rRNA genes. The *trn*L‐*trn*F region is located in the large single‐copy region of the chloroplast genome and includes the tRNA‐Leu (*trn*L) gene (partial sequence), the *trn*L‐*trn*F intergenic spacer (complete sequence), and the tRNA‐Phe (*trn*F) gene (partial sequence). The *trn*L‐*trn*F sequence of *S. malvifolia* is 876 bp in length, with a GC content of 36.9%. The entire nrDNA repeats, including ETS, 18S, ITS1, 5.8S, ITS2, and 26S regions, were 2199 bp in length. The GC content is 52.0%. The newly sequenced and newly assembled plastid genome, *trn*L‐*trn*F, and nrDNA sequences have been deposited in GenBank (accession numbers: PX570735, PX380933, and PX372082), and are listed in Table A1.

### 3.4 Molecular Phylogenetic Studies

The alignment of ITS sequences resulted in a matrix of 473 total characters, 277 of which are constant; 82 of the variable characters are parsimony‐uninformative, and 114 characters are parsimony‐informative. The alignment of *trn*L‐*trn*F sequences resulted in a matrix of 891 total characters, 701 of which are constant; 264 of the variable characters are parsimony‐uninformative, and 155 characters are parsimony‐informative. The best substitution models of the ITS and *trn*L‐*trn*F matrix were TIM2 + F + I + G4 and HKY + F + G4, respectively.

**FIGURE 3:**
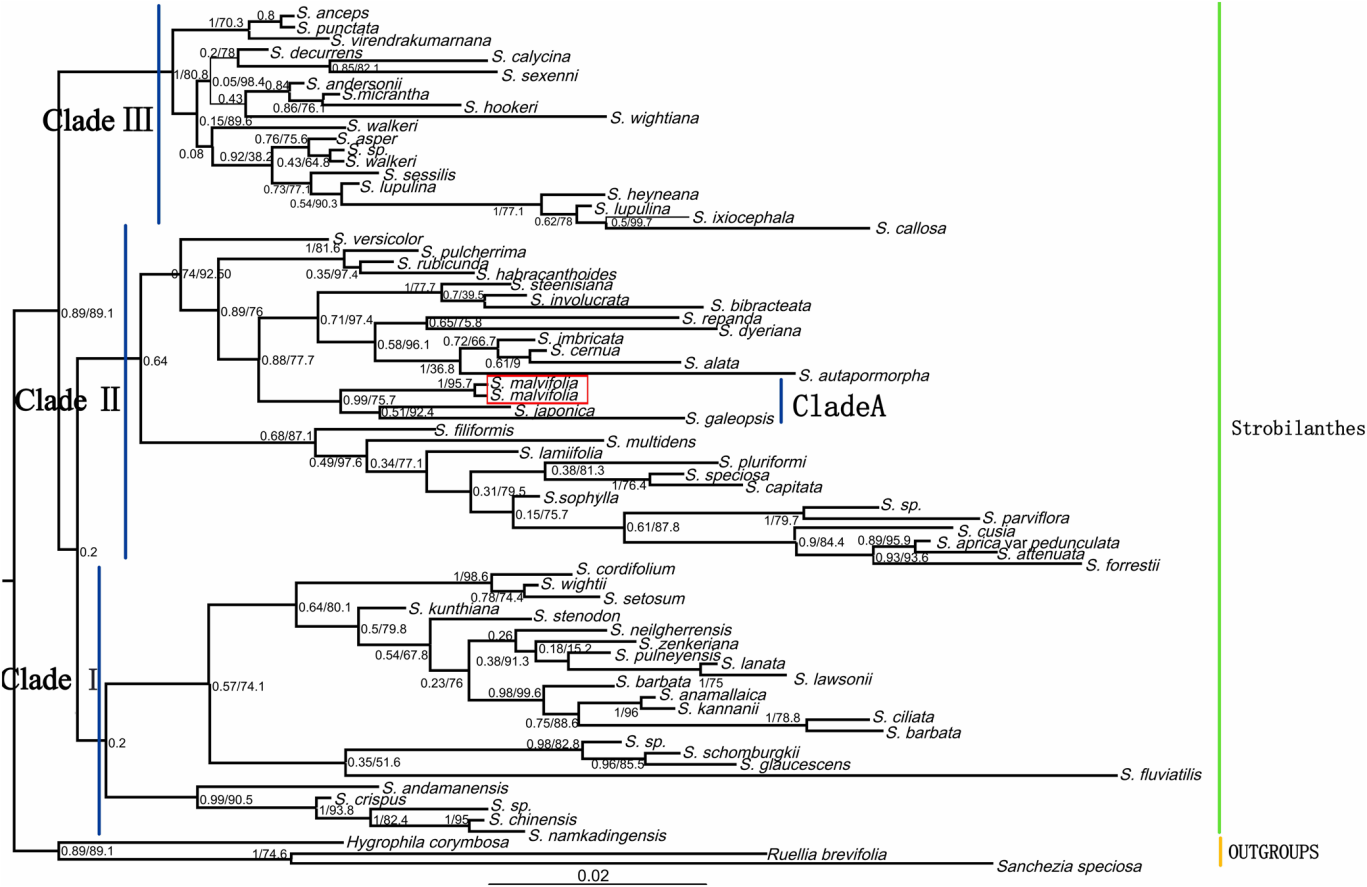
Phylogenetic tree of *Strobilanthes* Blume obtained by Mrbayes based on ITS sequences. Bootstrap percentages and Bayesian posterior probabilities are shown near the nodes (BI_PP_/BP_ML_). The position of *S. malvifolia* sp. nov. is marked by a red box.

**FIGURE A1:**
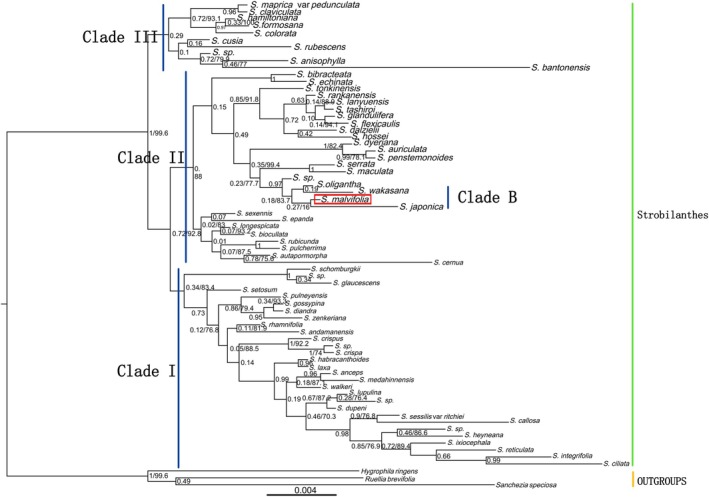
Phylogenetic tree of *Strobilanthes* Blume obtained by Mrbayes based on *trn*L‐*trn*F sequences. Bootstrap percentages and Bayesian posterior probabilities are shown near the nodes (BI_PP_/BP_ML_). The position of *S. malvifolia* sp. nov. is marked by a red box.

The present study reconstructed the phylogenetic relationships of the genus *Strobilanthes* using two methods (ML and BI) based on ITS and *trn*L‐*trn*F datasets, which were similar to the results of an earlier study (Moylan et al. 2004; Fernandes et al. 2025). The phylogenetic trees generated from the ITS and *trn*L‐*trn*F datasets showed that the topology of the Bayesian inference (BI) tree was largely congruent with that of the maximum likelihood (ML) strict consensus tree. Therefore, only the Bayesian strict consensus trees are presented in Figure 3 and Figure A1, with Bayesian posterior probabilities and maximum likelihood bootstrap values indicated.

**FIGURE A2:**
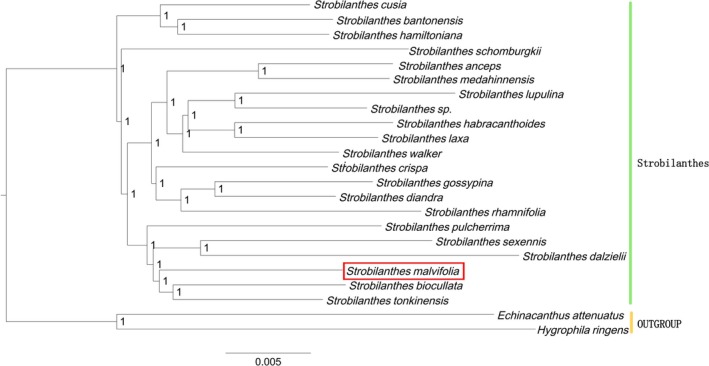
Phylogenetic tree of *Strobilanthes* Blume obtained by Mrbayes based on chloroplast genome sequences. The Bayesian inference posterior probabilities (BI_PP_) are indicated next to the nodes. The position of *S. malvifolia* sp. nov. is marked by a red box.

All sampled species sampled of *Strobilanthes* formed a well‐supported monophyletic clade in all analyses, and the majority of the subclades within the genus had strong support (Figure 3, BI_PP_ = 0.89/ML_BS_ = 89.1%; Figure A1, BI_PP_ = 1/ML_BS_ = 99.6%; all support values follow this order hereafter). The phylogenetic tree based on nuclear ITS sequences supported the division of *Strobilanthes* into three major clades (Clades I–III), with the relationship structured as ((Clade I, Clade II), Clade III) (Figure 3). Most species in Clade I are distributed in the tropical lowlands of the Indochina Peninsula—including Vietnam, Laos, Cambodia, Thailand, and Myanmar—with only a few exceptions such as *S. fluviatilis* (C. B. Clark ex W. W. Sm.) Moylan & Y. F. Deng and *S. chinensis* (Nees) J. R. I. Wood & Y. F. Deng occurring in southern China. Clade II contains numerous species distributed across a wide geographical range, including China, Japan, India, Malaysia, Thailand, and Java. As for Clade III, this clade comprises species with a relatively narrow distribution, confined to the Indian subcontinent (India and Sri Lanka), and it has not been recorded in China. Within Clade II, the newly discovered species *S. malvifolia* is recovered in a distinct sublineage (Clade A) and forms a strongly supported sister group with *S. japonica* (Thunb.) Miq. and *S. galeopsis* Stapf (Figure 3, BI_PP_ = 0.99/ML_BS_ = 75.7%). A plastid phylogeny constructed from *trn*L‐*trn*F sequences also supported the division of *Strobilanthes* into three major monophyletic clades (Figure A1, Clades I–III), in general agreement with the nuclear ITS phylogeny. However, discrepancies in the placement of certain taxa between the two analyses were observed, likely due to limited sampling and potential cytonuclear discordance. For example, *S. cusia* was placed in Clade II in the ITS phylogeny but grouped into Clade III in the *trn*L‐*trn*F phylogeny, suggesting possible cytonuclear incongruence or a complex evolutionary history. In the *trn*L‐*trn*F phylogeny, *S. malvifolia* is placed within Clade B, a relatively distinct lineage under Clade II, together with *S. japonica* , *S. oligantha* Miquel, and *S. wakasana* Wakas. & Naruh. in the same sublineage (Figure A1). Furthermore, although the phylogenetic tree constructed based on chloroplast DNA (cpDNA) has limited reference value for assessing the systematic position of this species due to the small number of available sequences, we have nevertheless included it in the appendix for reference (Figure A2).

## 4 Discussion

### 4.1 Morphological Distinctiveness of *Strobilanthes malvifolia*

**FIGURE 4:**
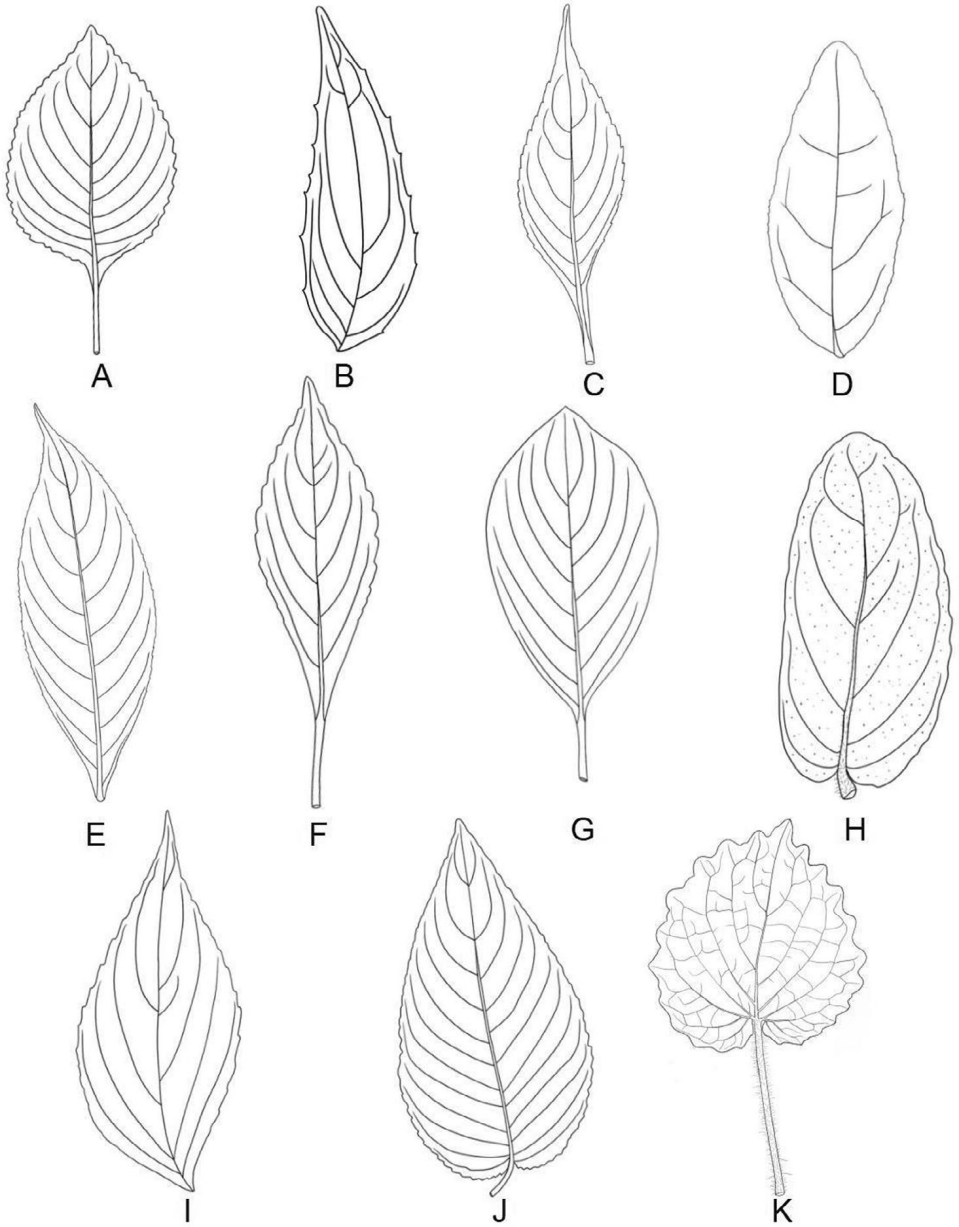
Leaf shape diversity of *Strobilanthes* (Drawn by Ting T. Wang). (A) *S. kingdonii* J. R. I. Wood. (B) *S. mastersii* T. Anderson. (C) *S. atropurpurea* Nees. (D) *S. japonica* (Thunb.) Miq. (E) *S. auriculata* Nees. (F) *S. henryi* Hemsl. (G) *S. aprica* (Hance) T. Anderson. (H) *S. reptans* (G. Forst.) Moylan ex Y. F. Deng & J. R. I. Wood. (I) *S. oligantha* Miquel. (J) *S. tomentosa* (Nees) J. R. I. Wood. (K) *S. malvifolia* sp. nov.

Leaf shape in *Strobilanthes* is highly variable, including lanceolate, ovate, elliptic, orbicular, and rhomboid forms, and serves as an important diagnostic feature for distinguishing species and infraspecific taxa (Figure 4) (Deng et al. 2006; Hu et al. 2011). However, among previously documented *Strobilanthes* species from China and adjacent regions, truly cordate leaves have rarely been reported. Only a few species, such as *S. attenuata* Jacq. ex Nees and *S. forrestii* Diels, have cordate leaf bases. In the strict sense, *S. malvifolia* represents the first species in the genus with cordate leaves (Figure 4). Its leaf blade shows a nearly 1:1 length‐to‐width ratio, and the opposite leaves are nearly equal in size. The petiole is relatively long, reaching up to 8.7 cm. In addition, the leaf margin of *S. malvifolia* is highly distinctive bearing sparsely distributed, irregularly crenate teeth, a trait that is also rarely seen in other *Strobilanthes* species.

We conducted a comprehensive comparative analysis of the major vegetative and reproductive characters of *S. malvifolia* and its phylogenetically related species ( *S. japonica* and *S. galeopsis*) as well as the morphologically more similar species ( *S. attenuata* ). The results are presented in Table 1, which clearly highlights the diagnostic traits that distinguish *S. malvifolia* from these related taxa. In terms of reproductive structures, *S. malvifolia* exhibits a series of stable and pronounced diagnostic characters. In addition to its markedly distinctive leaf characteristics, *S. malvifolia* also exhibits clear differences in stem morphology. Its stems are densely covered with long hairs and bear crinite and pilose indumentum, which sharply contrasts with the nearly glabrous stems of *S. japonica* , the fulvo‐tomentose stems of *S. galeopsis*, and the hirsute and setose stems of *S. attenuata* . In terms of reproductive structures, *S. malvifolia* likewise possesses stable and pronounced diagnostic features. The bracts are lanceolate and densely pubescent, the calyx lobes bear glandular and pubescent hairs and are nearly equal in length, and the corolla is light purplish‐red.

### 4.2 Phylogenetic Analysis of *Strobilanthes malvifolia*

Currently, there is no widely accepted classification system for the genus *Strobilanthes*. Bremekamp (1944) mainly based on pollen and seed morphology, divided the subtribe Strobilanthinae into 54 genera and 27 informal groups. Pollen morphology holds significant value in the phylogenetic studies of the family Acanthaceae, particularly in comparative studies among different genera and species (Bremekamp 1944; Deng et al. 2006; Lindau 1895). Compared to other members of Acanthaceae, the genus *Strobilanthes* exhibits a greater diversity in pollen morphology, which has been extensively applied in species delimitation (Carine and Scotland 2002; Wang and Blackmore 2003; Hu et al. 2011). Scanning electron microscopy (SEM) observations show that the pollen grains of *S. malvifolia* are globose, with wart‐like protrusions of various sizes on the exine, arranged in clear or indistinct rows. A comparison of pollen traits reveals that the pollen of *S. malvifoli*a closely resembles the type 10 identified by Hu et al. (2011), whose representative species is *S. tetrasperma* (Champ. ex Benth.) Druce. *S. tetrasperma* was classified in the Chamiponella group (Bremekamp 1944; Deng et al. 2006; Deng, Wood, and Fu 2010; Deng, Wood, and Li 2010). The group currently comprises 11 species: *S. austrosinensis* Y. F. Deng & J. R. I. Wood, *S. japonica* , *S. labordei* H. Lév., *S. lihengiae* Y. F. Deng & J. R. I. Wood, *S. longiflora* Benoist, *S. oligantha* , *S. sarcorrhiza* (C. Ling) C. Z. Cheng ex Y. F. Deng & N. H. Xia, *S. tetrasperma*, *S. wakasana*, *S. wilsonii* J. R. I. Wood & Y. F. Deng, and *S. yunnanensis* Diels (Li and Deng 2024). Through examining the pollen morphology of other species in the Chamiponella group (Deng et al. 2006; Gao et al. 2011), we found that *S. malvifolia* shares the same pollen type with members of this group. This similarity in pollen morphology is largely congruent with results of the phylogenetic analyses based on ITS and *trn*L‐*trn*F sequences.

Phylogenetic analysis based on ITS sequences revealed that *Strobilanthes malvifolia* and *S. japonica* form a clade (Figure 3, Clade A). Similarly, *S. malvifolia* and *S. japonica* continue to appear as sister taxa, and together form a clade (Clade B) with *Strobilanthes oligantha* Miq. and *Strobilanthes wakasana* Wakas. & Naruh. in the phylogenetic analysis based on the plastid *trn*L‐*trn*F sequence (Figure A1). Therefore, both phylogenetic evidence and palynological data support the placement of *S. malvifolia* within the Chamiponella group, indicating a close relationship with species such as *S. japonica* . Morphologically, *S. malvifolia* also matches key diagnostic characteristics of the Chamiponella group, including isomorphic petiolate leaves, terminal or axillary inflorescences, persistent bracts, nearly five‐lobed calyxes to the base, funnel‐shaped corollas, two strong fertile stamens, two ovules per ovary locule, and capsules containing four seeds. However, *S. malvifolia* is distinguished within the Chamiponella group by its unique leaf shape (cordate with sparsely and irregularly crenate margins) and habitat (dry‐hot river valleys), supporting its status as a distinct species within the group. Based on a comprehensive analysis of palynological, phylogenetic, and morphological data, *S. malvifolia* is most closely related to the Chamiponella group and is thus provisionally placed within this group of the genus *Strobilanthes*.

### 4.3 Habitat and Conservation Assessment

**FIGURE 5:**
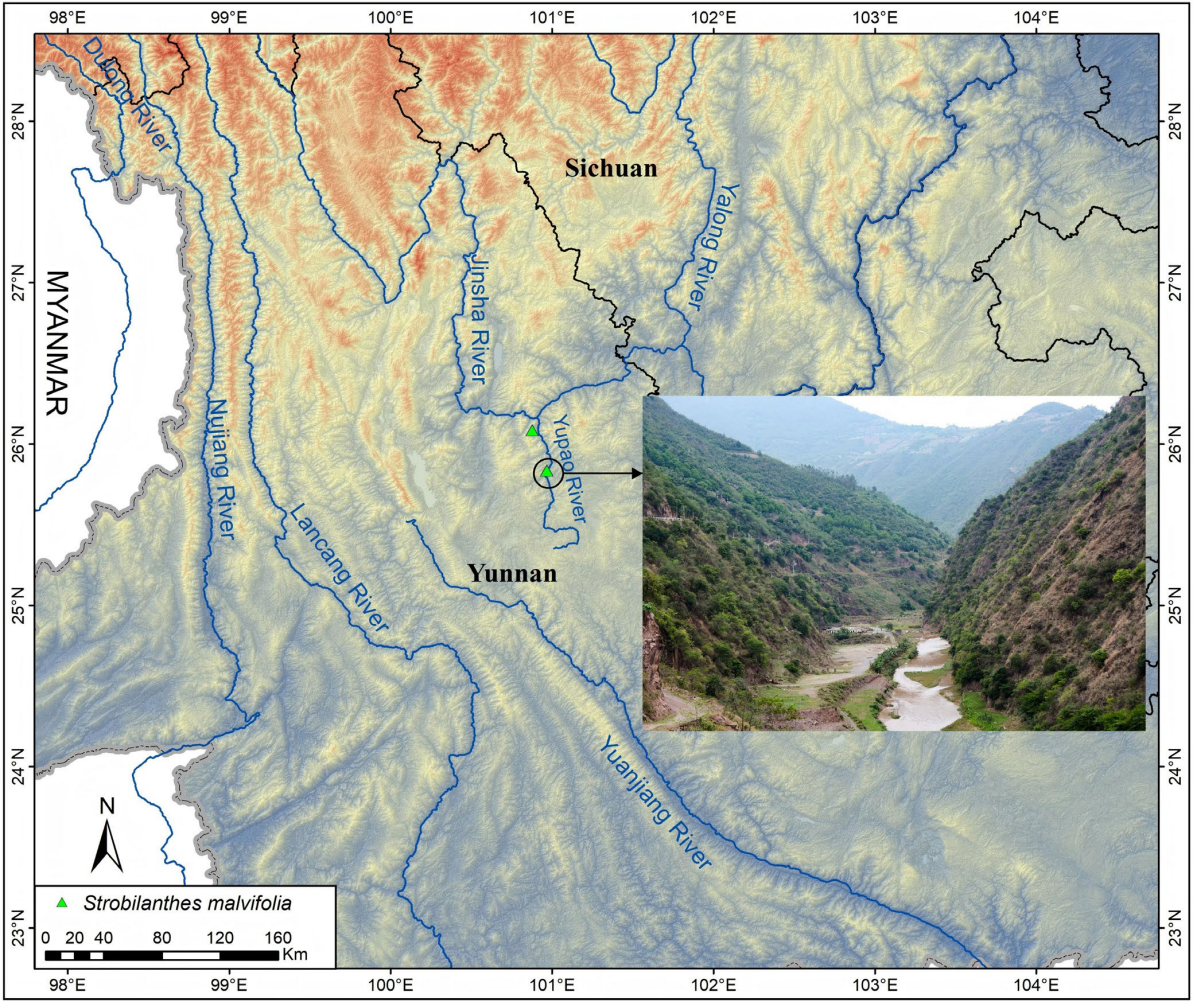
Geographical distribution of *Strobilanthes malvifolia* sp. nov. (green triangle).

Currently, *Strobilanthes malvifolia* is known from only two locations. One specimen (*W. M. Zhu et al. 02557*, YUKU) was collected approximately 60 years ago from Pingchuan Town, Binchuan County, Yunnan Province. However, our subsequent field investigations failed to relocate the species at this site. The other known locality is the type locality, located in Sanchahe Town, Dayao County, Chuxiong Yi Autonomous Prefecture, at an elevation of approximately 1500 m. Both distribution sites lie in the Yupao River valley of north‐central Yunnan Province (Figure 5). The Yupao River is a major tributary of the middle reaches of the Jinsha River. This region is characterized by deeply incised valleys and low‐lying terrain, with a typical dry‐hot valley climate (Zhang et al. 2013; Yang et al. 2016). The mean annual temperature ranges from 18°C to 22°C, with extremely hot summers and mild winters. Extreme maximum temperatures can exceed 38°C (Yang et al. 2021). Annual precipitation is 600–800 mm, with most rainfall concentrated between June and September. The area receives more than 2400 h of sunshine annually. Notably, the dry season lasts for 7–8 months, and the annual evaporation is approximately 3–5 times the precipitation. This significant moisture deficit results in long‐term soil stress, posing a severe challenge to plant survival. *S. malvifolia* mainly grows at elevations of 1400–1800 m in broad‐leaved forests or mixed coniferous and broad‐leaved forests dominated by *Pinus yunnanensis* Franch. (Pinaceae). It commonly occurs in forest understories and along forest edges, where the soil consists of mountain yellow soil or yellow‐red soil, and the bedrock is primarily limestone.

In southwest China, *Strobilanthes* exhibits remarkable species diversity (Hu and Cui 2006; Hu et al. 2011). In terms of habitat, most species grow in montane forests or shrublands and prefer warm, humid environments. Except for a few species, such as *S. henryi* Hemsl. and *S. tomentosa* (Nees) J. R. I. Wood, *Strobilanthes* species are rarely found in the dry‐hot valley habitats. A study by Nguyen et al. (2015) on *Strobilanthes* in Sri Lanka also showed that species diversity within the genus is significantly higher in moist, rainy areas than in arid regions. Other species in the Championella group are also widely distributed along shaded forest edges, valleys, or near streams in the southern to central subtropical regions of Asia, showing a clear preference for humid environments. Therefore, compared with other congeners, *S. malvifolia* exhibits a relatively unique adaptation to its habitat.

Based on current investigations, *Strobilanthes malvifolia* is a rare plant species with a very narrow distribution range, known only from its type locality and one nearby site. Fewer than 50 individuals have been recorded at its type locality, indicating a very small population size. The known distribution of this species is not included in any type of nature reserve or protected area, and the population is located near a rural road, making it highly susceptible to disturbances from road expansion and other human activities. According to the IUCN Red List Categories and Criteria (version 16) (IUCN 2024), this species meets the threshold for Criterion D (very small or restricted population), and we recommend it be preliminarily assessed as Endangered (EN).

## 5 Taxonomy Treatment

### 5.1 *Strobilanthes malvifolia* Huan C. Wang & Huai Yu Chen, sp. nov. (Figures 6, 7, 8)

Type. CHINA. Yunnan Province: Dayao County, Sanchahe Town, 25°50′ N, 100°58′ E, alt. 1500 m, 3 October 2023, (*S.Y. Liu et al. DY23106*) (Holotype YUKU!; isotypes YUKU!, PE!, KUN!).

**FIGURE 6:**
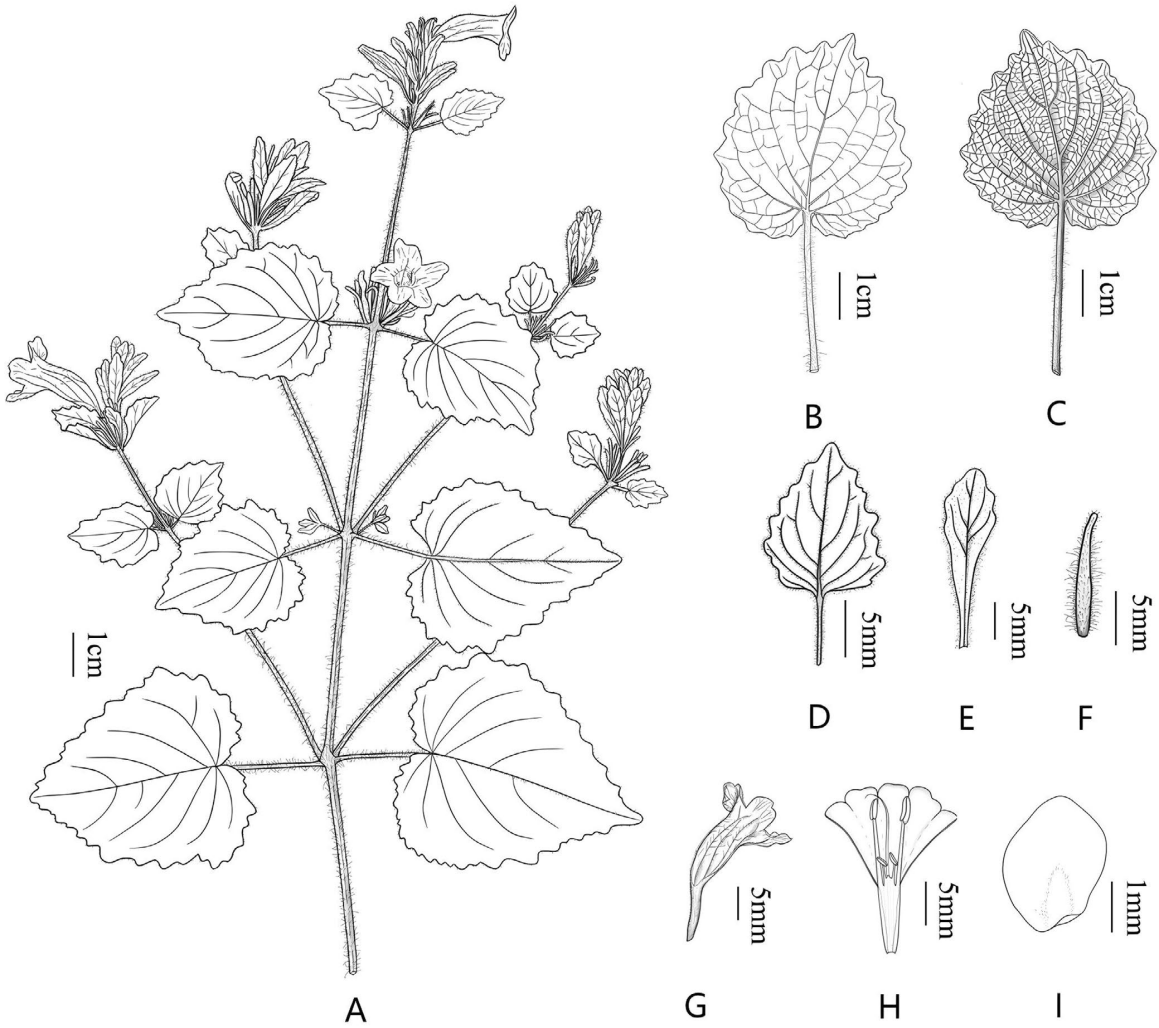
*Strobilanthes malvifolia* sp. nov. (Drawn by Ting T. Wang). (A) habit. (B) Adaxial surface of leaf. (C) Abaxial surface of leaf. (D) Lower bract. (E) Floral bract. (F) Bracteole. (G) Flower (side view). (H) Corolla dissected to show both androecium and gynoecium. (I) Seed.

**FIGURE 7:**
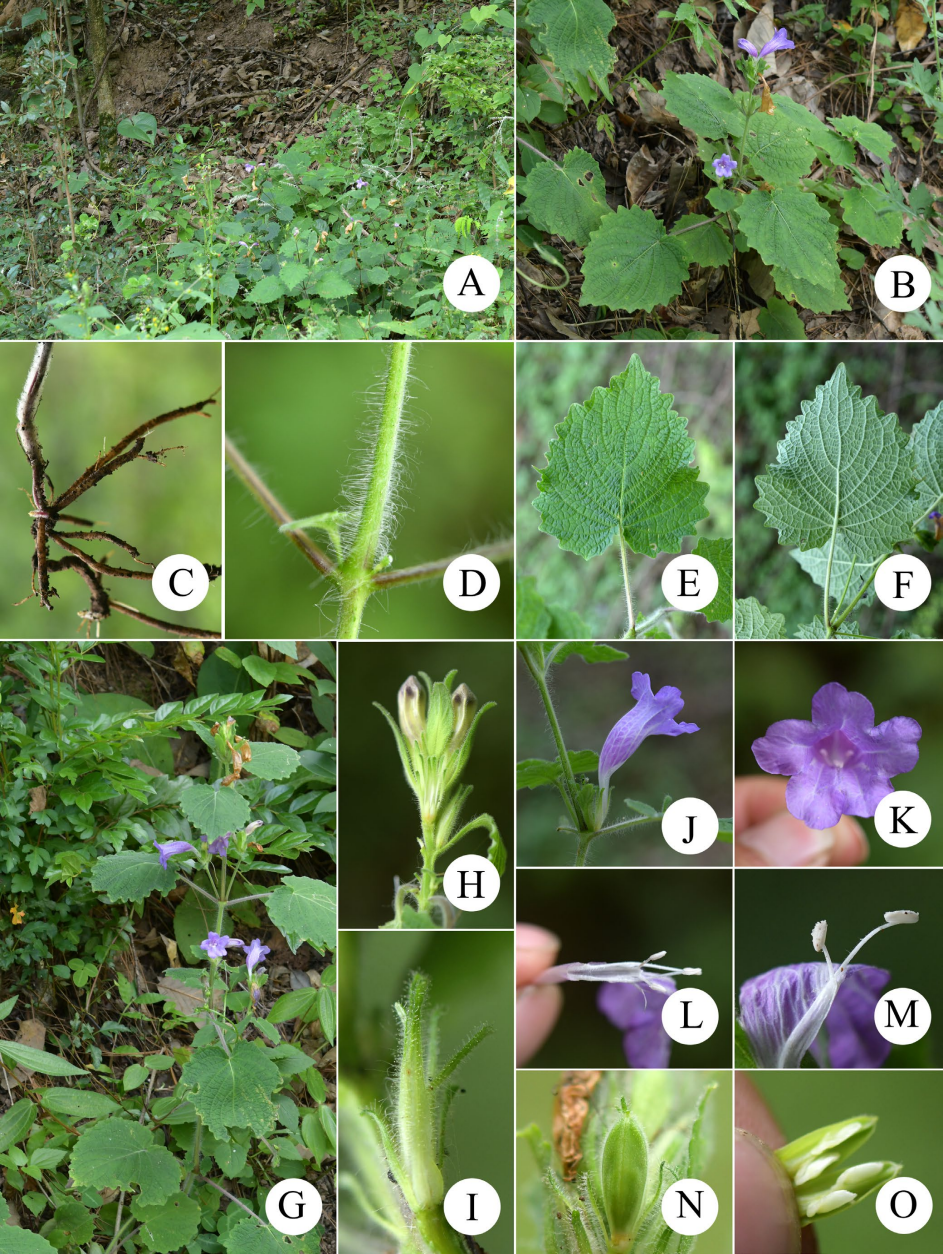
*Strobilanthes malvifolia* sp. nov. (A, B) Habit. (C) Roots. (D) A portion of stem, with densely villous. (E) Adaxial surface of leaf. (F) Abaxial surface of leaf. (G) Spike inflorescence on branches, forming a large panicle. (H) A portion of spike inflorescence to show bracteoles. (I) A calyx lobe. (J) Flower (side view). (K) Flower (front view). (L) Corolla dissected to show androecium. (M) Oblong anthers and curved filaments with pubescent. (N) Unmature fusiform capsule. (O) Opened capsule to show seeds.

**FIGURE 8:**
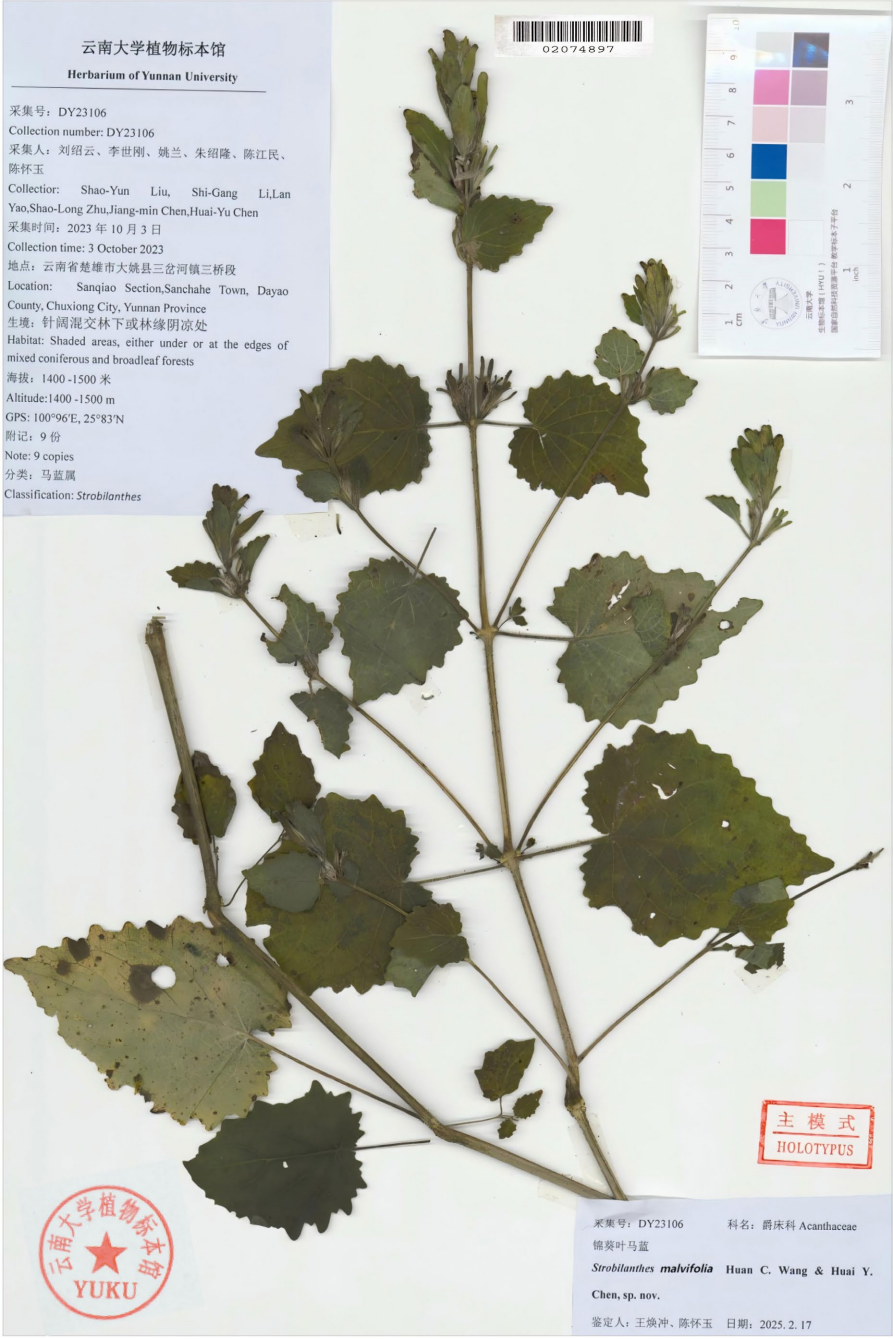
The holotype of *Strobilanthes malvifolia* sp. nov. (YUKU‐02074897).

### 5.2 Diagnosis

*Strobilanthes malvifolia* can be distinguished from other species of *Strobilanthes* by the following characters: leaves cordate, with a length‐to‐width ratio nearly 1:1, base cordate, margin irregularly and sparsely crenate, with petioles up to 8.7 cm long, densely spreading pilose; bracts variable in shape, including foliaceous, elliptic, ovate, or oblong forms, and persistent.

### 5.3 Description

Herbs perennial, 0.3–0.8 m tall. Rhizome, slightly elongated, fibrous roots. Stem single, erect, somewhat flexuose, base subterete, usually densely lanate; remainder quadrangular, channeled, densely crinite and pilose. Leaves decussate, cordate, isophyllous, 4–11 cm long, 3.5–10 cm wide, papyraceous; base cordate; apex acuminate; margin irregularly crenate (9–16 pairs); veins 4–7 pairs, adaxially impressed, abaxially prominent; surface green, veins densely crinite, remainder pilose; abaxial surface pale green, veins densely crinite, remainder sparsely pilose; petiole 1–8.7 cm long, densely spreading crinite. Spikes terminal or axillary, paniculate on upper stems, 3–12 cm long, bearing 2–10 flowers, flowers decussate; peduncle quadrangular, 2–12 cm long, glandular crinite. Lower bracts foliaceous, 1.5–3 cm long, 1–3 cm wide, penniveined, margin crenate, both surfaces densely piloglandulose, persistent; floral bracts long‐elliptic, ovate, or oblong, 2–3 cm long, 0.3–0.8 cm wide, persistent; bractlets two, linear or linear‐lanceolate, 0.5–1 cm long, both surfaces densely piloglandulose, margin entire, densely ciliate. Calyx deeply five‐lobed, lobes linear to narrowly lanceolate, nearly equal, apex rounded, 0.7–2 cm long, green, densely glandular‐pilose. Corolla light purplish‐red, funnelform, limb 5‐lobed, lobes suborbicular, nearly equal, apex slightly emarginate. Corolla tube slender‐cylindrical, gibbous upward, densely glandular‐pilose externally, with two rows of short pubescence internally. Stamens 4, included, didynamous, longer pair straight, shorter pair incurved, filaments pubescent, basally connate, anthers elliptic, two‐locular, longitudinally dehiscent, spurless; pistil solitary, stigma slightly exserted, style pilose above. Capsule fusiform, 0.8–1.3 cm long. Seeds 4, obliquely ovoid, flattened, circum‐areolar, villosulous.

### 5.4 Etymology

The specific epithet “*malvifolia”* is derived from the generic name *Malva* L. (Malvaceae Juss.) by adding the suffix “‐folia” (denoting shape or form), specifically chosen to highlight the leaf morphological similarities of this new species to some members of the genus *Malva*, such as *Malva cathayensis* M. G. Gilbert, Y. Tang et Dorr particularly. This includes cordate leaf blades with shallow lobes or serrations, palmate venation patterns typical of mallows, and soft, fine pubescence resembling those found in many *Malva* species.

### 5.5 Vernacular Name

Simplified Chinese: 锦葵叶马蓝; Chinese pinyin: Jǐn kuí yè mǎ lán.

### 5.6 Phenology

Flowering period: September—November; Fruiting period: October—November.

### 5.7 Distribution

*Strobilanthes malvifolia* is found in western Yunnan Province, southwest China. Based on current surveys, this species is known only from its type locality, Sanchaha Town, Dayao County, Chuxiong Prefecture, and the neighboring Pingchuan Town, Binchuan County, Dali Prefecture, both situated in the dry‐hot valley of the Yupao River, a major tributary of the Jinsha River. It is a rare plant typically restricted to dry‐hot valleys and exhibits a unique adaptation to environments under severe water stress. Morphologically, *S. malvifolia* is a perennial deciduous herb, with most parts of the plant densely covered in hairs, features that likely reflect its adaptation to arid conditions. The species occurs at elevations of approximately 1400–1800 m. In the type location, the associated plants mainly include *Pinus yunnanensis*, *Castanopsis delavayi* Franch. (Fagaceae), *Quercus variabilis* Blume (Fagacea), *Pistacia weinmanniifolia* J. Poiss. ex Franch. (Anacardiaceae), *Castanopsis orthacantha* Franch. (Fagaceae), *Dalbergia yunnanensis* Franch. (Leguminosae), *Bidens pilosa* L. (Compositae), *Gonostegia hirta* (Blume) Miq. (Urticaceae), *Ageratina adenophora* (Spreng.) R. M. King et H. Rob. (Compositae), *Oplismenus undulatifolius* (Ard.) Roemer et Schuit. (Gramineae), *Shuteria involucrata* (Wall.) Wight et Arn. (Leguminosae), *Diospyros dumetorum* W. W. Sm. (Ebenaceae), *Ageratum conyzoides* L. (Compositae), *Achyranthes bidentata* Blume (Amaranthaceae).

### 5.8 Additional Specimens Examined

*Strobilanthes malvifolia*. China. Yunnan: Dayao County, Sanchahe town, elev. ca. 1500 m, 26 October 2023, *S. G. Li et al. DY23333* (YUKU); Binchuan County, Pingchuan town, elev. ca. 1800 m, August 1965, *W. M. Zhu et al. 02557* (YUKU).

## Author Contributions

**Huai‐Yu Chen:** writing – original draft (equal). **Feng Yang:** writing – review and editing (equal). **Shao‐Yun Liu:** investigation (equal). **Ting‐Ting Wang:** visualization (equal). **Huan‐Chong Wang:** writing – review and editing (equal).

## Funding

This work was supported by the National Natural Science Foundation of China (grant no: 31960040) and the Second Tibetan Plateau Scientific Expedition and Research (STEP) program (2019QZKK0502).

## Conflicts of Interest

The authors declare no conflicts of interest.

## Data Availability

The DNA sequences generated in the present study have been deposited in the National Center for Biotechnology Information (NCBI) database. The accession numbers and the information on the voucher specimens are available in Table A1. The voucher specimens of the new species were housed in YUKU, KUN, and PE.

## Appendix A

**TABLE A1:** Scientific names, voucher information, and GenBank accession numbers (ITS, *trn*L‐*trn*F and cpDNA) of the specimens used in this study. A dash (−) indicates missing data.

	Species	ITS	trnL‐trnF	cpDNA
1	*Strobilanthes malvifolia*	PX372082	PX380933	PX570735
2	*Strobilanthes stenodon*	AY489372	—	—
3	*Strobilanthes neilgherrensis*	AY489373	—	—
4	*Strobilanthes pulneyensis*	AY489374	AY491719	—
5	*Strobilanthes lawsonii*	AY489375	—	—
6	*Strobilanthes wightii*	AY489378	—	—
7	*Strobilanthes setosum*	AY489379	AY491722	—
8	*Strobilanthes cordifolium*	AY489380	—	—
9	*Strobilanthes barbata*	AY489382	—	—
10	*Strobilanthes kunthiana*	AY489377	—	—
11	*Strobilanthes andamanensis*	AY489386	AY491724	—
12	*Strobilanthes micrantha*	AY489388	—	—
13	*Strobilanthes walker*	AY489391	—	—
14	*Strobilanthes anceps*	AY489395	—	MW411188
15	*Strobilanthes punctata*	AY489396	—	—
16	*Strobilanthes lupulina*	AY489397	—	MW802211
17	*Strobilanthes asper*	AY489399	—	—
18	*Strobilanthes decurrens*	AY489390	—	—
19	*Strobilanthes pulcherrima*	AY489368	—	MZ707510
20	*Strobilanthes habracanthoides*	AY489369	—	MZ707512
21	*Strobilanthes rubicunda*	AY489370	AY491710	—
22	*Strobilanthes galeopsis*	AY489354	—	—
23	*Strobilanthes japonica*	AY489356	AY491715	—
24	*Strobilanthes steenisiana*	AY489357	—	—
25	*Strobilanthes involucrata*	AY489358	—	—
26	*Strobilanthes bibracteata*	AY489359	AY491717	—
27	*Strobilanthes alata*	AY489360	—	—
28	*Strobilanthes cernua*	AY489361	AB161980	—
29	*Strobilanthes imbricata*	AY489362	—	—
30	*Strobilanthes repanda*	AY489366	AB161998	—
31	*Strobilanthes dyeriana*	AY489367	AB161985	—
32	*Strobilanthes filiformis*	AY489353	—	—
33	*Strobilanthes pluriformis*	AY489346	—	—
34	*Strobilanthes lamiifolia*	AY489347	—	—
35	*Strobilanthes multidens*	AY489350	—	—
36	*Strobilanthes speciosa*	AY489348	—	—
37	*Strobilanthes capitata*	AY489349	—	—
38	*Strobilanthes isophylla*	AY489352	—	—
39	*Strobilanthes forrestii*	JX443809	—	—
40	*Strobilanthes attenuata*	AY489344	—	—
41	*Strobilanthes aprica* var. *pedunculata*	AY489345	AY491706	—
42	*Hygrophila corymbosa*	KC420550	—	—
43	*Sanchezia speciosa*	OQ355470	AF063113	—
44	*Ruellia brevifolia*	EF214456	AB162025	—
45	*Strobilanthes ciliata*	MN381290	MN381329	—
46	*Strobilanthes barbata*	MN381288	—	—
47	*Strobilanthes virendrakumarana*	MT896160	—	—
48	*Strobilanthes callosa*	MN381289	MN381328	—
49	*Strobilanthes heyneana*	MN381291	MN381330	—
50	*Strobilanthes versicolor*	MT914278	—	—
51	*Strobilanthes walkeri*	MT889663	—	MZ707518
52	*Strobilanthes* sp.	MT896163	MN381337	MW802212
53	*Strobilanthes kannanii*	MT896162	—	—
54	*Strobilanthes ixiocephala*	MN381292	MN381332	—
55	*Strobilanthes lupulina*	MN381293	—	—
56	*Strobilanthes anamallaica*	MT876221	—	—
57	*Strobilanthes zenkeriana*	AY489371	AY491718	—
58	*Strobilanthes hookeri*	AY489389	—	—
59	*Strobilanthes lanata*	AY489376	—	—
60	*Strobilanthes sessilis*	AY489398	MN381336	—
61	*Strobilanthes crispus*	AY489383	AY491721	MW599987
62	*Strobilanthes calycina*	AY489392	—	—
63	*Strobilanthes* sp.	AY489341	AY491699	—
64	*Strobilanthes parviflora*	AY489342	—	—
65	*Strobilanthes* sp.	AY489343	AY491720	—
66	*Strobilanthes sexennis*	AY489393	—	MZ707514
67	*Strobilanthes wightiana*	AY489394	—	—
68	*Strobilanthes namkadingensis*	LC257985	—	—
69	*Strobilanthes chinensis*	AY489384	—	—
70	*Strobilanthes glaucescens*	AY489339	AY491702	—
71	*Strobilanthes schomburgkii*	AY489338	—	MZ707515
72	*Strobilanthes autapormorpha*	AY489365	AY491712	—
73	*Strobilanthes cusia*	KP092809	—	MG874806
74	*Strobilanthes* sp.	AY489385	AY491720	—
75	*Strobilanthes fluviatilis*	KP744348	—	—
76	*Strobilanthes andersonii*	MT571642	—	—
77	*Strobilanthes anisophylla*	—	AB161981	—
78	*Strobilanthes* sp.	—	AY491708	—
79	*Strobilanthes rubescens*	—	AY491709	—
80	*Strobilanthes colorata*	—	AB161983	—
81	*Strobilanthes formosana*	—	AB161988	—
82	*Strobilanthes claviculata*	—	AY491707	—
83	*Strobilanthes dupeni*	—	AY491723	—
84	*Strobilanthes integrifolia*	—	MN381331	—
85	*Strobilanthes reticulata*	—	MN381335	—
86	*Strobilanthes auriculata*	—	AB161982	—
87	*Strobilanthes penstemonoides*	—	AB161996	—
88	*Strobilanthes serrata*	—	AB161999	—
89	*Strobilanthes maculata*	—	AY491713	—
90	*Strobilanthes oligantha*	—	AB161995	—
91	*Strobilanthes wakasana*	—	AB162001	—
92	*Strobilanthes flexicaulis*	—	AB161987	—
93	*Strobilanthes rankanensis*	—	AB161997	—
94	*Strobilanthes glandulifera*	—	AB161989	—
95	*Strobilanthes lanyuensis*	—	AB161993	—
96	*Strobilanthes tashiroi*	—	AB162000	—
97	*Strobilanthes hossei*	—	AB161991	—
98	*Strobilanthes echinata*	—	AB161986	—
99	*Strobilanthes longespicata*	—	AB161994	—
100	*Hygrophila ringens*	—	KP744157	—
101	*Strobilanthes bantonensis*	—	—	MT576695
102	*Strobilanthes hamiltoniana*	—	—	MZ707511
103	*Strobilanthes medahinnensis*	—	—	MW411189
104	*Strobilanthes laxa*	—	—	MZ707517
105	*Strobilanthes gossypina*	—	—	MZ707513
106	*Strobilanthes diandra*	—	—	MZ707519
107	*Strobilanthes rhamnifolia*	—	—	MZ707516
108	*Strobilanthes dalzielii*	—	—	OR713747
109	*Strobilanthes biocullata*	—	—	MW044601
110	*Strobilanthes tonkinensis*	—	—	MW525447
111	*Echinacanthus attenuatus*	—	—	NC_039762
112	*Hygrophila ringens*	—	—	NC_080349
